# Evaluation des connaissances des parents sur les bronchiolites aiguës

**DOI:** 10.11604/pamj.2014.17.310.2717

**Published:** 2014-04-24

**Authors:** Widad Gueddari, Abderrahmane Tazi, Amine Ouardi, Samira Nani, Abdelhadi Zineddine

**Affiliations:** 1 Service d'Accueil des Urgences Pédiatriques, Hôpital d'Enfants Abderrahim Harouchi, CHU Ibn Rochd de Casablanca, Maroc; 2 Service d'Epidémiologie, Faculté de Médecine et de Pharmacie, Université Hassan II, Casablanca, Maroc

**Keywords:** Evaluation, connaissance, infection respiratoire, bronchiolite aiguë, nourrisson, Evaluation, knowledge, respiratory infection, acute bronchiolitis, infant

## Abstract

Les infections respiratoires (IR) constituent la deuxième cause de mortalité infantile au Maroc, dû en partie à l'absence d'information et de sensibilisation. Le but de ce travail était d’évaluer les connaissances des parents sur la bronchiolite aiguë, infection respiratoire très fréquente. Nous avons réalisé une enquête basée sur un questionnaire, auprès de parents de nourrissons consultants pour toux, avec ou sans gêne respiratoire. 180 parents ont été inclus dans l’étude. Les parents pensaient que l'infection respiratoire était secondaire au climat froid (96%); seuls 4% ont évoqué une origine infectieuse. Aucun des parents ne savait que le lavage des mains était un moyen de prévention de la transmission. Les parents ont majoritairement répondu que la kinésithérapie respiratoire ne servait à rien (65%), et qu'elle était nocive (24.5%). Ce manque de connaissances fondamentales en matière d'IR et de bronchiolite en particulier, devrait inciter à entreprendre un programme de sensibilisation

## Introduction

La bronchiolite aiguë est définie comme une infection des voies aériennes inférieures d'origine virale, survenant chez les nourrissons de moins de 24 mois. Elle n'est habituellement traitée que de façon symptomatique [1]. Le traitement spécifique antiviral, très couteux, n'est recommandé que chez les nourrissons ayant un terrain à haut risque de morbidité et de mortalité [2, 3]. Plusieurs études ont montré que le meilleur moyen thérapeutique est la prévention [4, 5]. Celle-ci ne peut se faire que par la sensibilisation et l'information des parents sur la maladie et les moyens de sa transmission, ainsi que sur les moyens thérapeutiques disponibles. Au Maroc, la sensibilisation de masse ne se fait presque pas. A l'inverse, dans d'autres pays comme en France par exemple, il y a des brochures et des sites internet pour sensibiliser les parents. Le but de ce travail était d'identifier le niveau des connaissances des parents au Maroc, en matière de bronchiolites aiguës des nourrissons.

## Méthodes

Sur une période de 5 mois allant du 1^er^ octobre au 31 mars 2012, nous avons réalisé une étude prospective au Service d'Accueil des Urgences Pédiatriques (SAUP) du centre hospitalier universitaire de Casablanca, à l'aide d'un questionnaire traduit en arabe et validé en interne. Les parents inclus étaient ceux dont les enfants ont consulté pour une bronchiolite aiguë, et qui ont accepté de participer de façon volontaire et éclairée à l'enquête. Le questionnaire était administré par un résident du service, sous la responsabilité d'un senior urgentiste pédiatre, immédiatement après la consultation et la prise en charge du nourrisson. Ont été exclus de l’étude les parents dont les nourrissons présentaient une bronchiolite grave. L'analyse descriptive des données a été faite à l'aide du logiciel Epi Info version 8.0.

## Résultats

Durant la période de l’étude, 220 nourrissons ont consulté au SAUP pour bronchiolite aiguë. Cent quatre vingt parents de nourrissons ont accepté de répondre au questionnaire. L'enquête a été faite dans 98% des cas avec la mère seule. Quatre vingt dix pour cent des mamans étaient des femmes au foyer. La moyenne d’âge des nourrissons était 9.7 ± 5,4 mois. Ils étaient gardés à la maison dans 92.3% des cas et dans une crèche dans 7.7% des cas. L'allaitement maternel était exclusif chez 28% des nourrissons pour une durée de 4 mois, et chez 19% des nourrissons pour une durée de 6 mois.

Les parents croyaient dans 96% des cas que la bronchiolite était secondaire au climat froid; seuls 4% des parents ont évoqué une origine infectieuse (« microbe »). Pour 147 parents, la bronchiolite était considérée comme une affection contagieuse. Ces mêmes personnes ont répondu que les moyens de transmission étaient l'air dans 69% des cas, le contact cutané direct dans 21% des cas; 10% ont répondu qu'ils ne savaient pas. Les mesures préventives utilisées selon les parents étaient: recouvrir correctement les bébés contre le froid (39%); éviter le contact avec les adultes enrhumés (31%); éviter les bains et douches (9.4%); utiliser de bavettes par les adultes enrhumés; 20.6% ont répondu qu'ils ne savaient pas. Les traitements administrés par les parents avant la consultation au SAUP étaient les antitussifs dans 56% des cas, l'huile d'olive dans 36% des cas, et les antibiotiques dans 34% des cas. Les parents ont répondu dans 65% des cas que la kinésithérapie respiratoire ne servait à rien et qu'elle était même nocive dans 24.5% des cas. Cent seize parents ont répondu qu'ils n'avaient aucune information sur les moyens préventifs de la bronchiolite et des infections respiratoires en général.

## Discussion

Cette enquête a révélé que la majorité des parents (96%) ne savent pas que l'origine de la bronchiolite est infectieuse. D'où leur ignorance concernant les moyens de transmission et une grande partie du traitement préventif. Pour les 117 parents qui ont répondu qu'ils utilisaient des mesures pour éviter la transmission de la maladie aux autres enfants, aucun n'a mentionné le lavage des mains, pourtant mesure préventive facile et efficace. Une étude française [6] basée sur l’évaluation des connaissances et des comportements des parents après la distribution d'une brochure sur la bronchiolite à la sortie de la maternité, a montré que le lavage des mains a été mentionné uniquement 10 fois sur 34. Dans notre étude, l'ignorance de cette mesure préventive par les parents peut être expliquée par le manque total d'information préalable sur la maladie et les moyens de sa transmission.

Dans notre population de nourrissons, le premier motif de consultation était la fièvre. Celle-ci est habituelle au cours d'une bronchiolite. Ce n'est que quand elle est élevée ou persiste qu'une surinfection bactérienne est suspectée, motivant la consultation d'un médecin. L'existence d'un seul hôpital d'enfants dans la ville de Casablanca, et peut être le manque de sensibilisation et d'information des parents, font qu'ils consultent directement au SAUP, au lieu de passer par les médecins de première ligne des hôpitaux régionaux ou ceux du secteur privé.

Notons concernant le traitement, l'utilisation importante des antitussifs (56%), suivie de l'huile d'olive (supposé être un fluidifiant bronchique) et des antibiotiques (prescrits par des médecins ou recommandés par les pharmaciens). On peut l'expliquer par l'absence de recommandations nationales concernant la prise en charge de la bronchiolite aiguë. De ce fait, les pratiques professionnelles sont divergentes et les parents suivent ce qui leur est dit. Pour 65% d'entre eux, la kinésithérapie, souvent prescrite par nos médecins, ne servirait à rien et serait même nocive pour 24.5% d'entre eux. Les méthodes utilisées par nos kinésithérapeutes sont disparates: nombreux sont ceux qui utilisent une sonde d'aspiration, sans accélération du flux expiratoire ni déclenchement du réflexe de la toux. Ces manœuvres sont vécues par les parents comme une torture, d'autant plus que les enfants crient et s'agitent. Sans compter le traumatisme de la muqueuse respiratoire qu'elles occasionnent.

La majorité des parents ont répondu qu'ils ne savaient pas comment éviter que les nourrissons, surtout les plus petits, ne soient atteints de bronchiolite. Ce résultat est prévisible, puisqu'ils n'ont eu aucune information concernant la maladie. L'allaitement maternel exclusif est connu comme moyen préventif des infections respiratoires chez les nourrissons. Vu que 90% des mamans ne travaillaient pas au moment de l'enquête, on se serait attendu à un nombre assez important de nourrissons sous allaitement maternel exclusif pendant les six premiers mois. Cependant, ce ne sont que 19% des nourrissons qui ont reçu le lait maternel exclusif pendant les six premiers mois et 28% pendant les 4 premiers mois. L'ignorance totale des bienfaits de l'allaitement maternel et l'absence de préparation des parents durant la grossesse à une bonne prise en charge de leur bébé à la naissance restent la seule explication à ce comportement.

## Conclusion

Il apparaît à travers cette enquête un manque évident de connaissances fondamentales chez les parents au Maroc, en matière de pathologie respiratoire en général et de bronchiolite en particulier. Un programme de sensibilisation à grande échelle devrait être entrepris, afin de contribuer à la diminution de la morbidité et de la mortalité liées aux infections respiratoires dans notre pays. Ce programme peut avoir comme support des brochures d'information, des émissions de vulgarisation à travers les médias, et même des visites dans les crèches pour sensibiliser et éduquer aussi bien les parents que le personnel qui s'occupe des nourrissons.

## Annexes

**Annexe 1 (PDF - 270 Kb):** Questionnaire pour l’évaluation des connaissances des parents sur les bronchiolites aiguës au Maroc
